# Current Role of Endoscopic Endonasal Approach for Craniopharyngiomas: A 10-Year Systematic Review and Meta-Analysis Comparison with the Open Transcranial Approach

**DOI:** 10.3390/brainsci13060842

**Published:** 2023-05-23

**Authors:** Luisa F. Figueredo, Andrea L. Martínez, Paola Suarez-Meade, Lina Marenco-Hillembrand, Andrés Felipe Salazar, Daniela Pabon, Juan Guzmán, Renata Murguiondo-Perez, Hana Hallak, Alex Godo, Carolina Sandoval-Garcia, Edgar G. Ordoñez-Rubiano, Angela Donaldson, Kaisorn L. Chaichana, María Peris-Celda, Bernard R. Bendok, Susan L. Samson, Alfredo Quinones-Hinojosa, Joao Paulo Almeida

**Affiliations:** 1 Department of Neurological Surgery, Mayo Clinic, Jacksonville, FL 32224, USA; lf.figueredo1341@gmail.com (L.F.F.); meade.paola@mayo.edu (P.S.-M.); marenco.lina@mayo.edu (L.M.-H.); chaichana.kaisorn@mayo.edu (K.L.C.); samson.susan@mayo.edu (S.L.S.); quinones-hinojosa.alfredo@mayo.edu (A.Q.-H.); 2 Faculty of Medicine, Universidad de Los Andes, Bogotá 111711, Colombia; al.martinez1433@gmail.com (A.L.M.); andresalazarab@gmail.com (A.F.S.); d.pabon10@uniandes.edu.co (D.P.); jd.guzman13@uniandes.edu.co (J.G.); 3 Faculty of Medicine, Universidad Anáhuac México Norte, Huixquilucan C.P. 52786, Mexico; renatamurguiondo@hotmail.com; 4 Department of Neurological Surgery, Mayo Clinic, Rochester, MN 55901, USA; hanahallak00@gmail.com (H.H.); periscelda.maria@mayo.edu (M.P.-C.); 5 Faculty of Medicine, Universitat Pompeu Fabra & Universitat Autònoma de Barcelona, 08002 Barcelona, Spain; alex.godo01@estudiant.upf.edu; 6 Department of Neurosurgery, University of Minnesota Medical School, Minneapolis, MN 55455, USA; csandova@umn.edu; 7 Department of Neurological Surgery, Fundación Universitaria de las Ciencias de la Salud, Hospital de San José—Sociedad de Cirugía de Bogotá, Bogotá 111711, Colombia; egordonez@fucsalud.edu.co; 8 Department of Otolaryngology (ENT), Head and Neck Surgery, Mayo Clinic, Jacksonville, FL 32224, USA; donaldson.angela@mayo.edu; 9 Department of Neurological Surgery, Mayo Clinic, Phoenix, AZ 85054, USA; bendok.bernard@mayo.edu; 10 Department of Internal Medicine, Division of Endocrinology, Mayo Clinic, Jacksonville, FL 32224, USA

**Keywords:** craniopharyngioma, microscopic transsphenoidal, endoscopic endonasal, systematic review, meta-analysis, transcranial

## Abstract

In recent years, the endoscopic endonasal approach (EEA) for craniopharyngiomas has proven to be a safe option for extensive tumor resection, with minimal or no manipulation of the optic nerves and excellent visualization of the superior hypophyseal branches when compared to the Transcranial Approach (TCA). However, there is an ongoing debate regarding the criteria for selecting different approaches. To explore the current results of EEA and discuss its role in the management of craniopharyngiomas, we performed MEDLINE, Embase, and LILACS searches from 2012 to 2022. Baseline characteristics, the extent of resection, and clinical outcomes were evaluated. Statistical analysis was performed through an X^2^ and Fisher exact test, and a comparison between quantitative variables through a Kruskal–Wallis and verified with post hoc Bonferroni. The tumor volume was similar in both groups (EEA 11.92 cm^3^, -TCA 13.23 cm^3^). The mean follow-up in months was 39.9 for EEA and 43.94 for TCA, *p* = 0.76). The EEA group presented a higher visual improvement rate (41.96% vs. 25% for TCA, *p* < 0.0001, OR 7.7). Permanent DI was less frequent with EEA (29.20% vs. 67.40% for TCA, *p* < 0.0001, OR 0.2). CSF Leaks occurred more frequently with EEA (9.94% vs. 0.70% for TCA, *p* < 0.0001, OR 15.8). Recurrence rates were lower in the EEA group (EEA 15.50% vs. for TCA 21.20%, *p* = 0.04, OR 0.7). Our results demonstrate that, in selected cases, EEA for resection of craniopharyngiomas is associated with better results regarding visual preservation and extent of tumor resection. Postoperative CSF leak rates associated with EEA have improved compared to the historical series. The decision-making process should consider each person’s characteristics; however, it is noticeable that recent data regarding EEA justify its widespread application as a first-line approach in centers of excellence for skull base surgery.

## 1. Introduction

Craniopharyngiomas (CP) are benign epithelial tumors that histologically arise from embryonic cell rests and present with solid, cystic, or mixed characteristics [1]. Treatment options include surgical resection, cyst aspiration with Ommaya reservoirs, radiation therapy, and, more recently, medical therapy. When surgical resection is selected, the choice between an endonasal endoscopic approach (EEA) or open transcranial approach (TCA) resection remains controversial [1,2].

Currently, EEA is applied in many centers as the approach of choice for pituitary adenomas and has also achieved a “gold standard” status for many suprasellar tumors, including CP. The development of high-quality visualization tools, such as 3D and 4K technology, and dedicated surgical instruments has improved the surgical resection of those tumors. Refinement of techniques for tumor resection and skull base reconstruction have positively impacted the results of the endoscopic CP surgery [3], likely leading to improved surgical outcomes in more recent years compared to the effects observed in previous decades (1990-the 2000s).

A recent meta-analysis showed better visual and endocrine outcomes (including panhypopituitarism and diabetes insipidus) for the EEA compared to TCA. Moreover, these results show an interesting tendency that favors EEA [4]; however, this study included only studies that directly compared the techniques, limiting the number of articles to eight and including 370 patients. Additionally, previous reviews, such as the one published by Komotar et al. in 2012, which included a higher number of studies, showed that the differences were minor [5].

This systematic review aimed to identify and review studies published in the last ten years, presenting the efficacy and outcomes of EEA and TCA for patients with CP. We hypothesize that previous concerns related to the EEA regarding postoperative cerebrospinal fluid (CSF) leaks and extent of resection (EOR) are now less significant and that EEA has achieved results that allow it to be considered the approach of choice for CP resection.

## 2. Materials and Methods

### 2.1. Data Sources and Searches

A systematic review of the literature was performed following the Preferred Reporting Items for Systematic Reviews and Meta-analysis (PRISMA) guidelines (http://prisma-statement.org/PRISMAStatement/PRISMAStatement.aspx, URL accessed on 11 April 2022). The protocol of this review was also registered in Inplasy with the registration number INPLASY202310045202310045 (https://doi.org/10.37766/inplasy2023.1.0045). We performed MEDLINE, Embase, and LILACS searches, including articles in English and Spanish from the last ten years (2011–2021). No restrictions were placed on study periods, age, or sample size. Search words “craniopharyngioma,” “endoscopic endonasal”, and “open transcranial” combined with terms such as “follow-up,” “recurrence”, “outcome”, and “complications” were used. The first search was conducted in December 2021. Articles were screened using RAYYAN software (https://rayyan.ai/, accessed on 11 April 2022) by title and abstract.

### 2.2. Data Extraction and Data Quality

#### Eligibility Criteria and Study Selection

Studies meeting the following criteria were included: (a) retrospective and prospective studies and (b) observational studies (i.e., cross-sectional, case–control, case series). The outcomes included visual outcomes (improvement, no changes, worsening), endocrinological outcomes (permanent diabetes insipidus and hypopituitarism), operative site infection, meningitis, cerebrospinal fluid leak, stroke, hemorrhage, and mortality. Studies that with the following criteria were excluded if they were determined to be: (a) case reports, (b) studies testing genetic disorders, (c) poster presentation abstracts without full-text availability, (d) systematic reviews, and (e) metanalyses.

Three reviewers (LF, DP, and LM) performed study selection independently and evaluated studies based on a two-step procedure. The first step was identifying titles and abstracts, including the relationship between microscopic transsphenoidal, endoscopic endonasal, or open transcranial approaches for CP and post-surgical outcomes (PM-LM). This step was performed using the software RAYYAN (https://rayyan.ai/, accessed on 11 April 2022). The potentially relevant articles were evaluated in a second step. A full-text review was performed by three reviewers (LF, HH, and JG) to determine whether the studies effectively reported the prevalence, incidence, or hazard ratio for the outcomes. A final review was performed (LF), addressing the experience of the senior author of the paper. The criteria included experience in one if not both, techniques of at least 10 years or a fellowship in skull base surgery or endoscopic endonasal approaches with at least 5 years of experience. This information was gathered from Scopus and the author’s affiliations. Any disagreement was solved by a fourth and fifth reviewer (EO and AG).

### 2.3. Outcomes

The primary outcomes of interest were an improvement and/or worsening of visual function from baseline, permanent diabetes insipidus (DI), and hypopituitarism. Secondary outcomes included CSF leak, meningitis, site infection, stroke, intracranial hemorrhage, seizures, tumor recurrence, and death related to the procedure. Hypothalamic involvement was sorted as suggested by Puget et al. [6] as follows: Grade 0, no hypothalamic involvement is detected; Grade 1, the hypothalamus is displaced by the tumor; Grade 2, hypothalamic involvement (the hypothalamus is no longer identifiable).

### 2.4. Data Extraction and Quality Assessment

Data extractions were classified into (a) article characteristics (year, type of publication, and country), (b) demographics (age and gender), and (c) treatment-specific variables (type of treatment, pre-surgical baseline, EOR, intraoperative complications, and post-surgical outcomes). The quality of the information of the eligible studies was compared using the Methodological Index for Non-Randomized Studies (MINORS) tool [7]. With this tool, the classification was: Items that scored 0, not reported; 1, reported but inadequate; 2, reported and adequate. However, the ideal global score is 16 for non-comparative studies and 24 for comparative studies. The Cochrane GRADE approach was used to assess the quality of evidence, grading the studies from very low to high quality in a 4-tiered system.

### 2.5. Statistical Analysis

Data from the individual studies were combined by cohort and compared between the groups. Statistical analysis of categorical variables was performed through an X^2^ and Fisher exact test, comparing three variables through a Kruskal–Wallis method and verified with post hoc Bonferroni analysis. The significance level was established at *p* < 0.05, and all the confidence intervals (CI) were set to 95%. The statistical analysis was performed using GraphPad Prism version 8.0.0 for Windows, GraphPad Software, San Diego, CA, USA, www.graphpad.com (accessed date 11 April 2022). The study adhered to the relevant sections of the Preferred Reporting Items for Systematic Reviews and Meta-analysis (PRISMA) guidelines.

### 2.6. Meta-Analysis

Results are presented as forest plots. Including only studies that compared vis-à-vis both interventions in the same center and where at least two of the authors were experienced in open transcranial or endoscopic endonasal. We employed a random-effect model to consider the possible clinical diversity and methodological variation between studies, as it assumes unequal variance between studies and distributes statistical weighting more conservatively. The I2 statistic was used to estimate the percentage of total variation across studies owing to heterogeneity rather than chance, with values >50% considered as substantial heterogeneity. All *p* values were 2 sided with significance set at *p* < 0.050. Statistical analysis was conducted with Rstudio (2020) RStudio: Integrated Development for R. RStudio, PBC, Boston, MA, USA URL http://www.rstudio.com/, accessed on 11 April 2022.

### 2.7. Quality and Bias Assessment

The quality of evidence was assessed using the Oxford Center for Evidence-Based Medicine-Levels of Evidence and Grading Recommendations, Assessment, Development, and Evaluation (GRADE).

## 3. Results

### 3.1. Included Studies and Patient Characteristics

**Figure 1:**
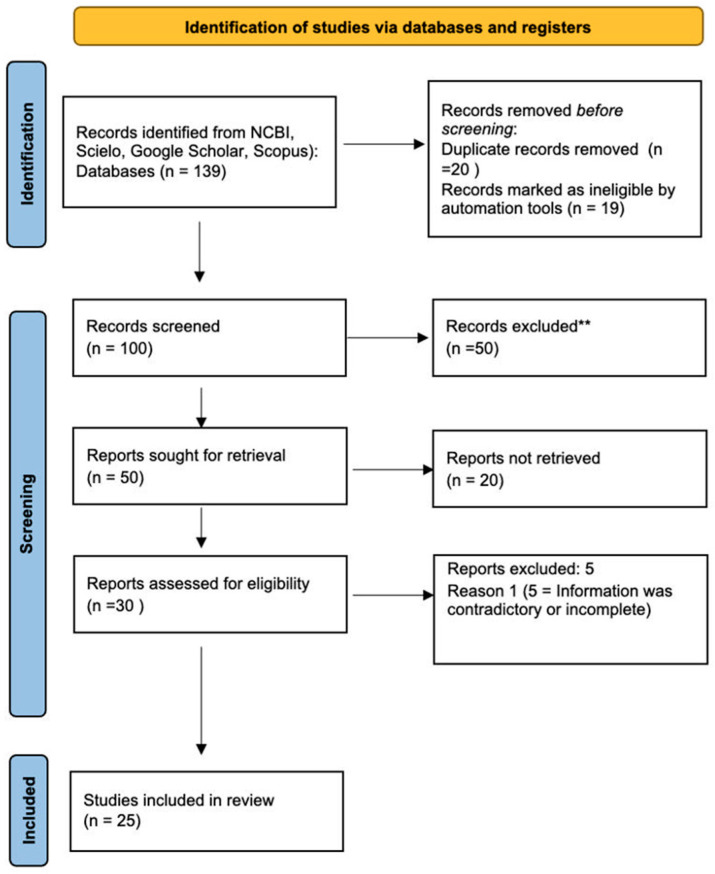
Flow diagram showing the process for study selection. ** not meeting the inclusion criteria.

In the end, 25 primary evidence articles were included in the review after a thorough evaluation. Of the initial 139 articles identified, 39 were duplicates, and 50 were excluded due to not meeting the inclusion criteria. The remaining 50 articles were evaluated by two reviewers, and 20 were excluded due to issues with the study quality, inconsistency in the results, or not differentiating between outcomes for patients who had both interventions. The details of the study selection process are summarized in Figure 1.

**Figure 2:**
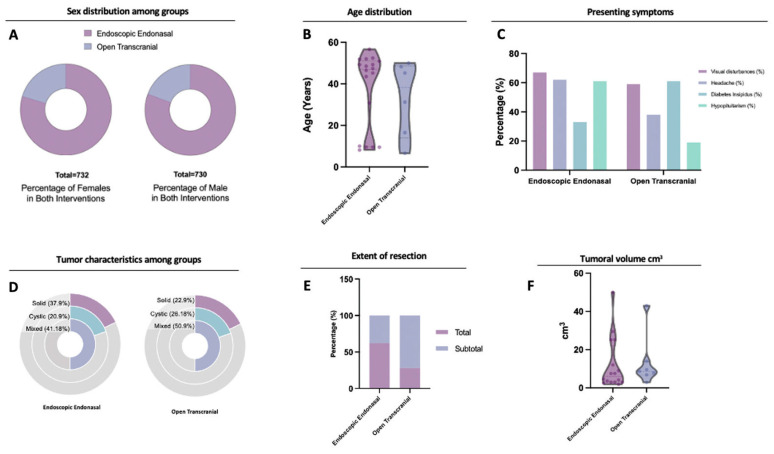
Comparison between EEA and TCA in (**A**) sex distribution between groups, showing a similar distribution and homogeneity in the comparison, and (**B**) age distribution between groups, showing a similar distribution. Note that a bimodal distribution in both groups is more noticeable in the EEA group. (**C**) Summary of presenting characteristics of the patients. Both groups’ most common initial symptom was visual disturbances (>50% in both interventions), followed by headache, hypopituitarism, and DI. (**D**) Tumor consistency. The most common presentation was mixed for both groups. (**E**) Gross total resection was more frequently achieved in the EEA group compared to the TCA, where most cases were managed via subtotal resection. (**F**) The endoscopic endonasal group showed a more extensive volume distribution than the TCA approach group; however, both groups’ mean distribution lies around similar volumes (<20 cm^3^).

A total of 25 studies were included, contributing to 1449 patients, this information is also summarized in Table 1. One thousand one hundred forty-four patients had EEA (78.9%) and 305 (21.1%) TCA (Table 2). Sex distribution was homogenous, with a percentage of females of 50.54% in the EEA group and 51.38% in the TCA groups (*p* = 0.84) (Figure 2A, Table 2). The mean age for the EEA group was 37.4 (CI 28–46.5), and for TCA was 37.1 (CI 13.9–52) (*p* = 0.96) (Table 2) (Figure 2B).

### 3.2. Clinical Presentation and Extent of Resection

The incidence of visual disturbances was higher in the EEA group (65.70%) compared to the TCA group (58.70%), with a significant difference of *p* = 0.03. Headaches were also more common in the EEA group (62.10% vs. 37.90% in TCA, *p* < 0.0001), while the incidence of DI was lower in the EEA group (30.7% vs. 61.1.1% in TCA, *p* = 0.005). Hypopituitarism was more prevalent in the EEA group (60.60% vs. 18.70% in TCA, *p* < 0.0001) (Figure 2C, Table 2). Mixed tumors (with solid and cystic components) were the most frequent type in both groups (41.18% in EEA and 50.90% in TCA, *p* = 0.83), followed by solid component tumors (37.91% in EEA and 22.9% in TCA, *p* = 0.71). Cystic tumors accounted for 20.92% in the EEA group and 26.18% in the TCA group (*p* = 0.61) (Figure 2D, Table 2). Mean tumor volume was similar in both groups, with 11.97 cm^3^ in the EEA group and 13.23 cm^3^ in the TCA group, with no significant difference (*p* = 0.83, 95% CI −11.53 to 14.05) (Figure 2F, Table 2).

Gross total resection (GTR) was achieved in 62.00% of EEA patients and in 28.17% of TCA patients (*p* = 0.62), while subtotal resection (STR) was more common in the TCA group (71.83% vs. 37.98% in EEA, *p* = 0.57) (Figure 2E, Table 2). The mean follow-up time for the EEA group was 39.9 months; for the TCA group, it was 43.9 months, with no significant difference (*p* = 0.76) (Table 2).

### 3.3. Surgical Outcomes

**Figure 3:**
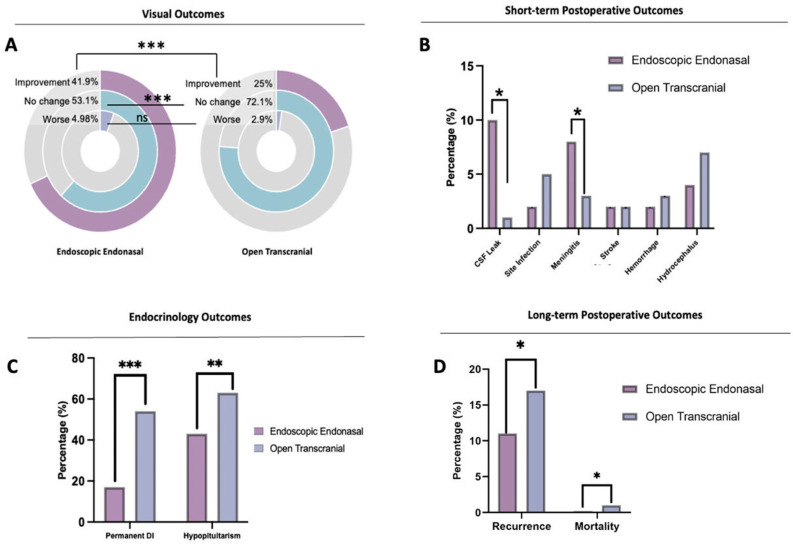
Summary of outcomes comparing EEA vs. TCA by visual outcome: (**A**) the graph shows a statistically significant improvement for the EEA group. In both groups, the visual symptoms worsened in less than 6% of the cases. (**B**) Summary of outcomes: CSF leaks occurred significantly more frequently in the EEA group; however, this happened in less than 10% of the total patients. Meningitis also occurred more frequently in the EEA group; this finding was also statistically significant. Other variables, including site infection, hemorrhage, and hydrocephalus, were more frequent in the TCA group. No differences were found in stroke. (**C**) Endocrinological outcomes, including permanent DI and hypopituitarism, occurred significantly more frequently in the TCA group. (**D**) A slight difference was found in mortality between the groups, being more frequent in the TCA. Recurrence as well was significantly more frequent in the TCA compared to EEA. * Statistically Significant, *p* < 0.05, ** *p* < 0.005, *** *p* < 0.001.

The EEA group had a higher rate of improvement in visual function from baseline compared to the TCA group (41.96% vs. 25.00%, respectively; *p* < 0.0001, OR 7.7, CI 5.4 to 11.1). There was no difference in worsening visual outcomes between the groups (*p* = 0.13) (Figure 3A) (Table 3). CSF leak was significantly more frequent in the EEA group (9.94%) compared to the TCA group (0.70%) (*p* < 0.0001, OR 15.8, CI 4.2–66.2) (Figure 3B) (Table 3). Site infection incidence was less than 5% in both groups (1.65% EEA, 4.57% TCA, *p* = 0.09, OR 0.35, CI 0.11–1.22). Meningitis occurred more frequently in the EEA group (8.12%) compared to the TCA group (2.55%), and this difference was statistically significant (*p* = 0.009) (Table 2) (Figure 3B). Seizures were only reported in the TCA group (1.71%) (Table 3). There was no significant difference in the incidence of stroke (Ischemic) between the groups (*p* = 0.98) (Figure 3B).

Permanent DI was more frequent in the TCA group (67.4%) compared to the EEA group (29.20%) (*p* < 0.0001, OR 0.2, CI 0.14 to 0.27). Similarly, post-operative hypopituitarism occurred more frequently in the TCA group (66.32%) than in the EEA group (46.80%) (*p* < 0.0001, OR 0.4, CI 0.33–0.58) (Figure 3C) (Table 3). The EEA group had a lower tumor recurrence rate than the TCA group (15.50% vs. 21.20%, respectively; *p* = 0.04, OR 0.7, CI 0.47 to 0.97). The mortality rate was similar in both approaches, at less than 3%, being less frequent in the EEA (0.77%) (*p* = 0.05, OR 0.9, CI 0.75 to 12.82) (Figure 3D) (Table 3).

### 3.4. Outcomes: Meta-Analysis

**Figure 4:**
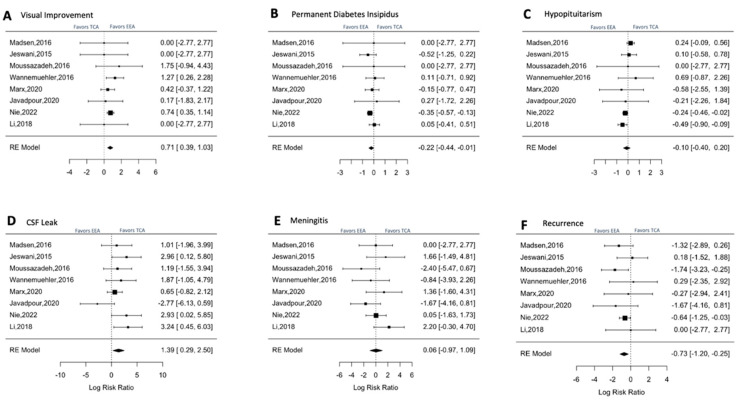
Meta-analysis of statistically significant outcomes from the systematic review analysis comparing endoscopic endonasal approach vs. open transcranial approach for craniopharyngioma management (Madsen, 2016 [32], Jeswani, 2015 [11], Moussazadeh, 2016 [12], Wannemuehler, 2016 [15], Marx, 2020 [25], Javadopour, 2020 [26], Nie, 2022 [29], Li, 2019 [30]). (**A**). Analysis comparing the rates of visual improvement between both interventions. (**B**). Risk evaluation of post-surgical permanent diabetes insipidus. (**C**). Risk evaluation of post-surgical hypopituitarism. (**D**). Risk evaluation of post-surgical CSF Leak. (**E**). Risk evaluation of post-surgical meningitis (all causes). (**F**). Comparison of recurrence rates.

Based on the analysis of the forest plot using a Random Effect model, there was a trend towards a higher rate of visual improvement in the endoscopic group, but that did not reach statistical significance (EEA and TCA groups (RR = 0.71, CI 0.39, 1.03, *p*-value 1.17) (Figure 4A) (Table 4). The Q-test indicated that the articles were heterogeneous but not significant as it falls into the 0–40% effect by Chi-square (Q = 3, *p* = 0.3, I2 = 29%). On the other hand, the risk of new-onset permanent DI was significantly reduced with EEA, as evidenced by a negative RR of −0.22 (CI −0.44, −0.01) (Figure 4B) (Table 4). For hypopituitarism, EEA also showed a negative RR of −0.10 (CI: −0.40, 0.20) (Figure 4C), indicating a trend favoring EEA. However, CSF leak was associated with an increased risk in the EEA group with a RR of 1.39 (CI: 0.29–2.5) and a *p*-value of 0.01 (Figure 4D) (Table 4). Lastly, the recurrence rate was significantly lower in the EEA group, with a RR of −0.73 (CI: −1.20, −0.25) (*p* = 0.002).

Of all articles, only four reported the location of the tumors in relation to the hypothalamus and overall changes in the follow-up that suggest hypothalamic disruption, including weight gain. Most articles that reported this outcome corresponded to pediatric cohorts and endoscopic endonasal approach, making using this information as a co-founder for the general analysis unsuitable. We summarize these results in Table 5. The range of new hypothalamic dysfunction lies between 4.4–17.5%. For these, the rate of GTR is low, being possible only in 30% of the cases in big cohorts [13,21,26,28].

## 4. Discussion

Although managing craniopharyngiomas (CP) can be complex, the endoscopic endonasal approach (EEA) is an excellent option for high-volume skull base centers of excellence. Advantages of EEA include higher rates of gross total resection (GTR), better visual and endocrinological outcomes, and less recurrence. However, the transcranial approach (TCA) also offers benefits, such as decreased cerebrospinal fluid (CSF) leak and meningitis rates.

CPs are benign tumors that originate from squamous epithelial remnants of the Rathke’s pouch and can form anywhere from the nasopharynx to the hypothalamus. These lesions are relatively rare, accounting for only 1% to 5% of all intracranial tumors in the US [13]. However, the prevalence appears to vary by age and geography. For instance, the prevalence is 7.9% in Europe and 11.6% in Africa [30].

Historically, CP resection was performed using microscopic transcranial approaches, including subfrontal, frontolateral, orbitozygomatic, and pterional routes. However, in the last 20 years, the EEA has provided a less invasive option with the potential for similar positive outcomes [31,33]. This is supported by the growing number of EEA publications in the last decade, as evidenced by the NCBI trends with a peak after 2017 for the keyword “Endoscopic Endonasal for Craniopharyngiomas”.

Existing indications for TCA instead of EEA include large suprasellar lesions with significant lateral extensions into the Sylvian fissure, encasement of the middle cerebral artery and lenticulostriate branches, lesions with peritumoral edema, and invasion of the brain parenchyma [34].

### 4.1. Presenting Symptoms

It was noted that patients that underwent an EEA presented with more visual disturbances and headaches prior to surgery (Table 2, Figure 2C). Preoperative DI was more frequent in the TCA group (39.10%), and hypopituitarism was more common in the EEA group (60.60%) (Table 2, Figure 2C).

### 4.2. The Extent of Resection

The extent of resection is a critical factor that significantly affects surgical outcomes. However, in the studies reviewed, the information on EOR was heterogeneous. Despite this, gross total resection (GTR) was significantly higher in endoscopic endonasal surgery (EEA), with 62.00% of patients achieving it, compared to 28.17% in transcranial approaches (TCA) (Table 2). It is important to consider that TCA approaches may have been selected for more challenging cases with a neurovascular encasement or hypothalamic extension and, therefore, presented a lower rate of GTR [13,14,25]. Another argument is that the decision to use TCA resulted from the tumors being bigger. However, the rate of visual symptoms, headache, and hypopituitarism pre-surgery indicate that the tumors resected by EEA may have been just as challenging.

Previous studies have shown that achieving GTR is still challenging due to the aggressive nature and invasiveness of CP, and it is an independent predictor of tumor recurrence [27,31]. The size and anatomical location are among the most important variables to consider when deciding on the approach. Giant CPs (>4 cm in diameter) are associated with higher neurological, endocrine, and hypothalamic morbidities during the postoperative period [1]. Generally, suprasellar lesions located medial to the neurovascular plane, i.e., carotid artery and optic nerves, are good candidates for EEA, whereas lesions with lateral extension into the Sylvian fissure or significant cranial extension beyond the foramen of Monro into the lateral ventricles are usually better approached via TCA [35]. There is some controversy on how to approach purely intraventricular craniopharyngiomas. Most surgeons favor transcranial approaches for intraventricular craniopharyngiomas that have not transgressed the floor of the third ventricle (“intact third ventricle floor”), but some centers have recently advocated for EEA even in those cases [36]. The concern with endoscopic approaches in those cases is related to potential injury to the hypothalamus during the opening of the floor of the third ventricle [19,37,38]. In cases where there is a clear transgression of the third ventricle floor, there is less controversy, and approach selection is often based on a lateral extension of the tumor, relationship with neurovascular structures, and surgical team experience [37,38].

It is worth noting that the improvement of visualization and angled endoscopic instruments has allowed further expansion of EEA in the coronal plane, and, therefore, selected craniopharyngiomas with lateral extension may be adequately approached via EEA, especially if mostly cystic [10]. In cases where complete tumor resection is not achieved, adjuvant radiation therapy, in the form of fractionated radiation therapy, radiosurgery, or proton beam therapy, may be considered. These therapies may lead to improved disease control rates [39]. Subtotal resection in combination with radiotherapy has been proven to be a good alternative for pituitary axis preservation, although prevention of recurrence is under debate [27].

The location of the CP and its correlation with outcomes may be subject to bias. There are different ways to classify CP, with the three most commonly used being (1) anatomical, (2) preoperative based on hypothalamic involvement, and (3) endoscopic based on the infundibulum topography [33,39]. Other classifications, including the one used by Hu et al., divided CP into three categories, Q, S, or T. Q CPs originated below the diaphragmatic area, Type S CP [33].

The studies included in this review presented a wide heterogeneity in the choice of classification and the description of the outcomes associated with the craniopharyngioma location, so the statistical analysis is less accurate and at high risk for type II error. However, some studies provide important results based on the anatomical characteristics of CP, such as the papers by Radovanovic et al., Lei et al., and Cavallo et al. Radovanovic used the Pittsburgh classification based on the relationship with the infundibulum, a widely used classification proposed by Kassam et al. in 2008 [34]. Their results showed that type III CP (Retroinfundibular, IIIa including third ventricle, or IIIb including the interpeduncular cistern) was associated with the lowest rates of GTR and the highest for recurrence after EEA [31]. However, there is no information regarding visual or endocrine outcomes based on the location.

Lei et al. used an anatomical classification (intrasellar, intra-suprasellar, suprasellar, intra-third ventricle). In their results, GTR was obtained in more than 80% of all locations, with the highest recurrence rate in the suprasellar type (7.9%) and intra-third ventricle (7.7%). The lowest recurrence and highest GTR were obtained in the intrasellar type (4%). Visual improvement was widely obtained in the intrasellar type (87.5%) and intra-suprasellar (65%) [35]. Finally, Cavallo et al. report higher rates of GTR in tumors located prechiasmatic (72.4%) or retrochiasmatic (80%). In mixed locations, pre-retrochiasmatic (being more extensive tumors) GTR corresponded to 40%. This group also included results regarding the CP location in relation to the stalk, where GTR was obtained in a 76.5% infundibular position, preinfundibular (69.6%), retroinfundibular (47.4%), and mixed location (72%). Finally, GTR of tumors with a compromise of the third ventricle with floor compression was achieved in 64% of the patients and in those with intraventricular extension 71.8%; however, data are scarce regarding outcomes (such as visual and endocrinological outcomes) and tumor relationship with the stalk [3].

### 4.3. CSF Leak, SITE Infection, and Meningitis Risk

Early retrospective cohorts and metanalysis reported CSF leaks in the EEA in 20% to 30% [5]. The report by Komotar et al. describes a CSF leak rate of 18.4% after EEA (*p* < 0.003). More recent studies from 2021 reported improved rates ranging from 3% to 15.9% [36]. Such differences are likely to reflect the advancement of endoscopic skull base surgery in the last 10 to 15 years, secondary to improvement in skull base reconstruction with techniques based on vascularized tissue.

In our results, we found an overall postoperative CSF leak rate of 9.94% in EEA cases, compared to 0.70% in the open TCA group (*p* < 0.0001, OR 15.8 CI: 4.2–66.2). Despite the higher rate of CSF leaks after EEA, there has been a significant decrease in the occurrence of such complications compared to previous reports. Several technical changes have been fostering the progress of the EEA, reducing complication rates. The most crucial factor was the introduction of the vascularized septal flap in 2007 [36,37] and other skull base reconstruction techniques, such as the gasket seal and button graft techniques [38,39]. Further improvements have also played a role, such as better quality endoscopes and instruments [40] and widespread knowledge of the endoscopic anatomy of the skull base and its applications in the tumor resection and reconstruction [41]. Regarding site infection and meningitis risk, our study found a significant increase in the risk for meningitis in the EEA approach (Table 4), likely associated with the higher chance of CSF leak in this group when compared to the transcranial group.

### 4.4. Endocrinologic Outcomes

Postoperative DI and hypopituitarism were most common in the TCA group (Table 2, Figure 4C). Similar results for postoperative hypopituitarism were reported by Komotar et al. favoring the EEA (*p* < 0.003). Metanalysis of this variable (Table 3, Figure 4C) demonstrated a decrease in the risk with EEA (RR of −10 (CI: −0.40, 0.20)). Some of the confounding factors that can influence the variability of results regarding endocrine outcomes include tumor size, EOR, and treatment with radiotherapy. More extensive tumors, especially those directly arising from the infundibulum, are likely to present hormonal dysfunction after surgery. Considering the complex nature of CP and its tendency to recur, one of the accepted treatment strategies is to proceed with gross total tumor resection when possible and to transect the pituitary stalk if necessary to achieve this goal [42]. This technique maximizes tumor control but leads to hormonal deficits and the need for a life-long hormonal replacement [8,20,31,40,41,42,43]. Endoscopic technique, with its high-quality visualization, can allow for the preservation of the pituitary stalk and its vasculature while achieving GTR in selected cases. Anatomical preservation of the pituitary stalk has been followed by physiological preservation of hormonal function in over 50% of cases [20].

### 4.5. Stroke, Hemorrhage, and Hydrocephalus

Our study indicates that the incidence of stroke, hemorrhage, and hydrocephalus related to the surgical procedure tended to be higher with the TCA than EEA but did not reach significance. EEA demonstrated a result of 2.10% for stroke, while TCA was 2.12%. For hemorrhage, EEA showed 2.30% and TCA 3.21%. Finally, EEA showed a result of 3.50% for hydrocephalus, while TCA showed 6.50%. In a previous systematic review by Komotar et al., 2.9 % of patients who underwent TCA presented with stroke (Ischemic) as a complication compared to 2.7% for EEA [2]. This difference was not statistically significant and is consistent with small differences among most studies. Some authors had previously expressed concern about the difficulty of controlling intraoperative hemorrhages during endoscopic skull base surgery. It remains easier to prevent vascular injuries with transcranial microsurgical techniques. However, there has been significant development of dedicated endoscopic skull base instruments, including dissectors and bipolar and surgical clips, that allow for a much-improved vascular control compared to earlier eras with EEA. Additionally, one of the advantages of EEA is its superior visualization of the surrounding vasculature, including superior hypophyseal arteries and perforating branches, which may lead to a lower chance of intraoperative arterial complications. Independently of the selected approach, microsurgical techniques, with a combination of sharp and dull dissection, should be applied via microscope or endoscopic visualization. Extensive use of curettes, lack of identification of arachnoid planes, and premature pulling of the tumor capsule should be avoided as they can lead to intraoperative vascular injuries.

Hydrocephalus was among the least frequent complications for both surgical approaches; some authors were more concerned about hydrocephalus as a pre-existing condition than a surgical complication [44]. However, Cagnazzo et al. presented results suggesting hydrocephalus was more common after the transsphenoidal endoscopic procedure [45]. Komotar et al. also showed that EEA carried a higher incidence for this complication, at 15.80% compared to 10.10% for TCA.

### 4.6. Recurrence and Mortality

CPs are typically known for having a high recurrence incidence after surgery. Our study indicates a higher recurrence rate after TCA when compared to EEA (Table 4**).** The TCA group showed recurrence rates up to 21.20% and 0.77% in mortality, while EEA showed recurrence in 15.50% and mortality rates of 2.22%, respectively. Moussazadeh et al. performed a single-institution retrospective study that found that EEA was associated with a decreased incidence of recurrence (*p* < 0.001), likely due to the higher rates of GTRs obtained with this procedure [9]. Patel et al. found that a recurrence rate of 22.60% in EEA was associated with a lower quality of life. However, they also found that the quality of life of individuals with recurrence was higher when treated with radiation rather than a second operation [7]. On the other hand, Komotar et al. reported a recurrence rate after TCA in 28.20% (*p* < 0.001) [5]. Many studies saw this trend for higher recurrence rates in the TCA [4]. A possible explanation for the worse outcomes in TCA could be inherent to the tumor characteristics and some undisclosed factors that influenced the decision to use TCA over EEA; however, the increased visual symptoms, headache, and hypopituitarism pre-surgery would hint that the tumors resected by EEA were just as challenging.

As for mortality, in our cohort, we found a prevalence of 0.77% for the EEA, meanwhile for the TCA group was 2.22% (*p* = 0.2); however, this number was not stratified in most of the descriptions by cause and was not clear in some of the studies if mortality was perioperative, postoperative, or due to specific endocrinological complications. Komotar et al. found that the perioperative mortality with the TCA was 3.20%, while it was 1.90% with EEA. However, this difference was not statistically significant [25]. This trend was also seen in a study conducted by Schelini et al., where they found that the mortality rates for patients subjected to the TCA are higher than EEA, with the results being 2.50% and 0.00%, respectively [22].

### 4.7. Hypothalamic Involvement

As mentioned in the results section, only four studies reported the location of the tumors in relation to the hypothalamus and overall changes in the follow-up that suggest hypothalamic disruption, including weight gain. Most articles that reported this outcome corresponded to pediatric cohorts and endoscopic endonasal approaches, making the use of the information as a co-founder for the general analysis unsuitable. However, the hypothalamic compromise plays a key role in the extent of the resection [11]. Postoperative obesity is a well-document sequela of craniopharyngioma resection, most likely due to hypothalamic injury. Jeswani et al. mentioned the difficulty addressing the weight status of each patient, besides the hypothalamic compromise, having other contributing factors, including steroid use, change of physical activity, and diet. For Leng et al., expanded endoscopic endonasal is a suitable approach in Puget Grade 1 cases, attempting a GTR. Tumor debulking via open transcranial is recommended for Grade 2, usually associated with anterior cerebral artery encasement, followed by fractionated RT [8]. In the Patel et al. case series, rates of new or worsening obesity were seen in 37.5 of patients; however, the majority of this cohort received GTR, making the researchers unable to comment on whether GTR or STR with RT had different rates of hypothalamic dysfunction [10]. Finally, as was presented by Hong and Omay in 2022, the modern surgical approach to craniopharyngiomas has been focused on maximally safe resection while minimizing postoperative complications, particularly in regard to sparing the optic nerves and hypothalamus. Compared to GTR, subtotal resection (STR) followed by adjuvant radiation has demonstrated similar rates of local control and has even been suggested by some groups to be the preferred treatment strategy [41].

### 4.8. Perspective on Medical Treatments for Craniopharyngiomas: BRAF-Targeted Therapies

The discovery of distinct oncogenic molecular signaling pathways in CP has opened a wide range of alternatives for personalized treatment [46]. The key pathogenic event in the adamantinomatous craniopharyngiomas (ACP) pathway corresponds to Wnt activation along with the alterations of the MEK/ERK pathway. In contrast, in papillary CP (PCP) there is primary activation of the MEK/ERK pathways by *BRAF*-V600E mutations [47,48]. Particularly for PCP, many clinical trials have been conducted since 2016. Some of the preliminary results in small cohorts suggest that the most effective therapy should include at least two agents, reducing the tumoral volume by almost 83%, as was described by Brastianos et al., in the Alliance A071601 trial [49], and in some other cohorts like the one presented by Calvanese et al. in 2022, describing a reduction in the tumoral volume of 90% after five months. Some of the current agents include Dabrafenib, Trametinib, and Vemurafenib. Juvatli et al. suggested that the concomitant use of Dabrafenib and Trametinib is probably the most effective in their six patients’ cohort with a reduction of 80–90% tumoral volume [47]. A limitation to these agents is that, even though they seem highly effective, the mutation is seen in 95% of adults setting PCP; 5% of the population still counts only in traditional management, which it makes pivotal to offer the best option for this group.

### 4.9. Limitations

This systematic review and meta-analysis have a number of limitations. Randomized studies are absent, and only clinical series are available; however, this limitation is intrinsic to the nature of the surgical interventions. There also was heterogeneity in the included studies, particularly concerning the reported outcomes, which were based on varied classifications for infundibular or hypothalamic involvement. As a result, the reported numbers were not appropriate for accurate statistical analysis, with a high risk for type II error. However, we presented a brief summary of the reported results, which were also reviewed in the discussion section.

A specific comparison between the imagenological characteristics of the tumors that made the researchers decide on one approach over the other was not performed due to the limited availability of the studies, as well as addressing medical treatment directed to *B-RAF* mutation as a cofounder. A variable that we recommend being addressed in future studies.

Additionally, intrinsic to a systematic review, obtaining a maximal number of high-quality information comes with the risk of including articles from centers with low case volumes with different levels of expertise among surgeons. However, to mitigate this possible bias, for the meta-analysis, we only included articles that compared vis-à-vis both interventions in the same center, and we checked that at least two of the authors were experienced in open transcranial or endoscopic endonasal. The Q-test indicated that the included studies in the metanalysis were heterogeneous but not statistically significant as the Chi-square falls into the 0–40%. Moreover, we advocate that prospective studies elaborate on the effect of exact preoperative characteristics in terms of the tumor’s location in relationship with the hypothalamus, involvement of the optical component (optic nerve and chiasm), and matched characteristics in size.

## 5. Conclusions

EEA is associated with higher rates of gross total resection and lower rates of postoperative hypopituitarism and permanent DI compared to TCA. Some trends also suggest lower rates of recurrence and mortality and higher rates of visual improvement. However, CSF leak remains one of the most significant concerns, although this complication rate has improved over time. Both surgical alternatives are safe, and the selection from one technique compared to the other should be tailored to the patient and the tumor anatomy, hormonal function, and previous treatments, with patient-specific goals established for each surgical case.

## Figures and Tables

**Table 1:** Summary of included studies.

Author	Year of Publication	Type of Study	Total Number of Patients	Total Follow-Up (Months)	Number of Patients with Endoscopic Resection	Number of Patients of Open Transcranial Resection
Leng et al. [8]	2011	Case Series	24	35	24	0
Ali et al. [9]	2013	Retrospective cohort	7	6.3	7	0
Patel et al. [10]	2015	Single Institution Review	31	40	31	0
Jeswani et al. [11]	2015	Single Institution retrospective Review	87	34.9	53	34
Moussazadeh et al. [12]	2016	Single Institution Review	26	30	21	5
Bal et al. [13]	2016	Case Series	25	56.4	25	0
Fomichev et al. [14]	2016	Case Series	136	10	136	0
Wannemuehler et al. [15]	2016	Retrospective review	21	7.2	9	12
Pennacchietti et al. [16]	2016	Retrospective review	104	18	104	0
Patel et al. [17]	2017	Cross-sectional	16	56.2	16	0
Nagata et al. [18]	2018	Retrospective review	12	41	0	0
Ishikawa et al. [19]	2018	Case Series	178	6	178	0
Forbes et al. [20]	2018	Prospective Cohort	10	46.8	10	0
Yamada et al. [21]	2018	Clinical series	65	93.6	65	0
Schelini et al. [22]	2019	Case Series	20	63.6	20	0
Massa et al. [23]	2020	Case Series	30	42.7	30	0
Pak-Yin Liu et al. [24]	2020	Retrospective cohort	28	60	0	28
Marx et al. [25]	2020	Retrospective cohort	30	136	17	13
Javadpour et al. [26]	2020	Prospective database	15	74	15	0
Mazzatenta et al. [27]	2020	Cross-sectional	25	72	25	0
Fomichev et al. [14]	2016	Retrospective Cohort	136	42	136	0
Cavallo et al. [28]	2014	Case Series	103	48	103	0
Nie et al. [29]	2022	Case Series	273	30.5	88	185
Li et al. [30]	2018	Retrospective cohort	43	9	17	26
Radovanovic et al. [31]	2019	Case Series	43	56.8	43	0

**Table 2:** Baseline characteristics of the patients by the intervention.

	Endoscopic Endonasal	Open Transcranial	*p* Value
Patient Characteristics			
Total Patients	1144	305	0.1
Mean Age (Range)	37.4	37.1	0.96
Female (%)	50.54	51.38	0.84
Male (%)	49.46	48.62	0.85
Mean follow-up (months)	39.9	43.9	0.76
Presenting Symptoms			
Visual disturbances (%) *	65.70	58.70	0.03
Headache (%) *	62.10	37.90	<0.0001
Diabetes Insipidus (%) *	30.70	61.10	0.005
Hypopituitarism (%) *	60.60	18.70	<0.0001
Tumor characteristics			
Volume (cm^3^)	11.97	13.23	0.83
Solid (%)	37.91	22.91	0.71
Cystic (%)	20.92	26.18	0.61
Mixed solid and cystic (%)	41.18	50.90	0.83
Extent of resection			
Gross Total (%)	62.00	28.17	0.62
Subtotal (%)	37.98	71.83	0.57

* Statistically significant.

**Table 3:** Postsurgical outcomes comparing endoscopic endonasal approach vs. open transcranial.

	Endoscopic Endonasal (%)	Open Transcranial (%)	*p* Value	OR	LCI	SCI
Visual Outcomes						
	41.96	25	<0.0001	7.7	5.4	11.1
No change *	53.06	72.09	<0.001	0.4	0.28	0.55
Worse	4.98	2.91	0.14	0.13	0.83	4.9
Endocrine Outcomes						
New Onset Permanent DI *	29.20	67.40	<0.0001	0.2	0.14	0.27
New Onset Hypopituitarism *	46.80	66.32	<0.0001	0.4	0.33	0.58
Postoperative Outcomes						
CSF Leak *	9.94	0.70	<0.0001	15.8	4.2	66.2
Site Infection	1.65	4.57	0.09	0.35	0.11	1.22
Meningitis *	8.12	2.55	0.009	3.38	1.59	7.62
Stroke	2.10	2.12	0.98	0.98	0.39	2.5
Hemorrhage	2.30	3.21	0.59	0.71	0.24	2.1
Hydrocephalus	3.50	6.50	0.4	0.5	0.15	1.77
Follow-Up						
Recurrence *	15.50	21.20	0.04	0.7	0.47	0.97
Mortality *	0.77	2.22	0.05	0.9	0.75	12.82

LCI: Lower Confidence Interval, SCI: Superior Confidence Interval. * Statistically Significant.

**Table 4:** Meta-analysis of statistically significant variables.

	EEA (Cases/Control)	TCA (Cases/Control)	*p*-Value	RR	LCI	SCI
Visual Outcomes						
Improvement	72/78	43/172	1.17	0.71	0.39	1.03
Outcomes						
Permanent DI	86/79	182/88	0.04	−0.22	−0.44	−0.01
Hypopituitarism	100/76	189/96	0.5	−0.10	−0.4	0.20
CSF Leak	31/181	2/288	0.01	1.39	0.29	2.50
Meningitis	6/163	7/268	0.9	0.06	−0.97	1.09
Recurrence	20/175	56/208	0.002	−0.73	−1.20	−0.25

**Table 5:** Summary of findings regarding hypothalamic involvement [13,21,26,28].

Article	Number of Patients (No Previous Intervention)	Mean Age	Type of Intervention	Classification of Hypothalamic Involvement	Number of Patients with Hypothalamic Involvement	Pre-operatory Hypothalamic Dysfunction	Post-operatory New Hypothalamic Dysfunction	Extent of Resection in Cases with Hypothalamic Involvement	Postoperativs Radiation	Post-Operative Main Symptom
Bal, 2016 [13]	15	31.2 (17–68)	EEA	Not Discussed	4	50% (2/4)	0%	GTR (50%)	Not Discussed	Not Discussed
								STR (50%)		
Yamada, 2018 [21]	45	9.6 (0.8–17.9)	EEA	Grade 0 = 19	26	4.4% (2/45)	4.4% (2/45)	GTR (98%) *	1	Weight Gain
				Grade 1 = 8				STR (2%)		
				Grade 2 = 18						
Javadopour, 2021 [26]	15	10 (5–18)	EEA	Grade 0 = 9	6	20% (3/15)	6.6% (1/15)	STR (100%)	6	Weight Gain
				Grade 1 = 0						
				Grade 2 = 6						
Cavallo, 2014 [28]	103	50.36 (18–83)	EEA	Not Discussed	25	24.3% (25/103)	17.5% (17/97)	GTR (30%) STR (70%)	8 patients	Not Discussed

* No difference was discussed of the extent of resection in patients with hypothalamic involvement only.

## Data Availability

All the data used in this work is already included in the manuscript.
